# Practical Observations and Suggestions in Medicine

**Published:** 1845-04

**Authors:** 


### PRACTICAL OBSERVATIONS AND SUGGESTIONS IN MEDICINE. By *Marshall Hall,* M.D. F.R.S. L. and E. &c. &c. 8vo. pp. 360. Churchill. London, 1845.

FEW men have been more active and stirring in their generation than the author of the present volume. At an early period of life, he entered the field of authorship; and, since the date of his first work, he has continued to keep his name before the public eye by a series of clever and ingenious productions. Of late years more especially, he has been unusually prolific. Since the first announcement of the Doctrine of the Reflex Functions, there has been an almost uninterrupted succession from his pen of papers, memoirs, books and lectures on the subject. One cause indeed of this "magna copia *scribendi*" is, that Dr. Hall is evidently, by nature as well as by taste, a controversialist. Impatient of contradiction, and with an inordinate appetency for praise, he never lets an opportunity pass of answering the objections, exposing the errors, and—sometimes too—of impugning the motives of whosoever may differ from him. He is always ready for the combat; and his weapons are keen, and adroitly—although too often uncourteously—handled. Even in the present volume—which, by the bye, is entitled "*Practical* Observations and Suggestions in Medicine"—the captious spirit sometimes oozes out. On one occasion, we read "that those discoveries (in regard to the functions of the spinal marrow) have been continually and very groundlessly called into question and made the subject of dispute, and that by gentlemen totally unprepared for entering upon that discussion;" and, on another, the whole profession is roundly reproved for having received one of our author's propositions with lukewarmness.

Dr. Hall has most of the characteristic attributes of the out-and-out reformer of fifteen years ago. Quick, clever, censorious and self-complacent, he is full of himself, and has *his* remedy ready for every difficulty. He is fond of *new-ness* in almost every thing; and so smitten is he with the love of innovation, that many old things he calls *new.* We need not say that the faculty of Veneration is not predominant in his character; and that he has no great respect, on the whole, for long-established opinions and practices. Hence it comes that he has little pleasure in dwelling upon the labours of others, except in so far as they serve to exalt the value of his own: he seldom expatiates at any length upon what has been done before his time; his thoughts are mainly occupied with the doings of himself and of his cotemporaries. He keeps an ever-wakeful eye on everything that is going on around him, and his alert mind is always busy in devising some new change or another. He does not seem to know, or at all events to appreciate, what artists mean by the term "repose." There is a restlessness about him that he cannot subdue; all is stirring and fermenting within and around him. Without the least imputation of vanity, he may very fairly affirm that "*his* has been a really active professional life." It would have been the same whatever had been the station in which he was placed, or the vocation to which he had applied himself. In humble life, he would have raised himself from the ranks to the post of adjutant, or perhaps even to the command of a company; from the toilsome drudgery of a village schoolmaster to the envied distinction of a clever sectarian preacher. The energy of his character is truly admirable; and would command universal respect, if it were not for the overweening egotism (shall we use a word that he himself has supplied us with? —*ego-mania*) that oft excites a smile, when it does not provoke a sneer: this it is which mars so grievously much that he has done, and done too so well. The first personal pronoun abounds, to a most alarming extent, in all his writings. He cannot wait for others to praise him; so that, every now and then, he is obliged to indulge in a little self-laudation. Not satisfied with telling us—in the retrospective notice of his authorship with which he has favoured us—that he has "allowed few days to elapse without recorded observation," he proceeds to remark, with a most *naive* complacency, "this habit *I* regard as the *test* of a physician's steadiness and industry." *My* "New Memoir" is characterised as containing "the most lucid and recent view of the whole subject of the Physiology and Pathology of the True Spinal System." But perhaps the most amusing instance of the dominant passion, is when our author sits down in the critic's chair, and takes upon himself to weigh, in the balance of practical importance, the comparative merits of his own professional labours. First and foremost of all are "*my* researches in regard to the use and abuse of blood-letting:" these, he goes on to say, "*I* look upon with the greatest satisfaction." In a previous publication, he very modestly pronounces the following judgment on his discovery of the effects of blood-letting in the erect posture, as a means of distinguishing inflammatory from irritative disease—a point of practice, by the bye, which has any thing but received the sanction of the profession. "There is, in *our* opinion, no single fact in physic of equal importance and value, in the diagnosis of acute diseases and the use of an important remedy." We are told, in reference to another of his diagnostic discoveries, "that it is one of great practical importance, and that it has proved the means of saving many dear and valuable lives;" and, after enumerating a few more instances of the benefits which he has done, he sums up his claims to public gratitude by saying, with the most graceful diffidence, "one other service which *I think* (the Italics are ours) I have rendered the art of medicine, in its application to infants and children, is that which relates to the treatment of infantile convulsions."

So all-absorbing does this habit of self-laudation seem to have become with our author, that he candidly admits that he has been led *almost unconsciously* into the enumeration of his own merits: we quite believe it. Would that, for his own sake, he would learn not to think more highly of himself than he ought to think; that he would leave to others the task—and it is an agreeable one—of praising his sayings and doings; and that he would be less impatient of censure, and less uncharitable towards those who differ from him. If he had taught himself, in early life, to feel the full force of that memorable saying, *Magna est veritas et prevalebit*, and had been willing to leave the results of his labours to the public for their award, he might, we are assured, have occupied a higher position in the scientific world; for his mind—unquestionably one of no common stamp—instead of being so often distracted by petty contentions and personal strifes, would have been left to pursue its trains of original enquiry in greater composure and with a more equally-sustained energy of thought. To be a great physician, a man must be of a calm and quiet mood; averse alike from the heats of controversy, and from the pursuit of any pet doctrines or favourite notions. Dr. Hall is unfortunately far from being exempt from these defects; and thus it has come that, on the whole, he is considered a better physiological than a practical writer: he is too fond of ingenious theories and too much smitten with the love of innovation to be an unexceptionable guide in the treatment of disease. Though he lived a thousand years, he could never become an Abercrombie or a Chambers.

While perusing the "Observations and Suggestions," we have on several occasions been reminded of Dr. Holland's "Medical Notes;" but this has been rather in the way of contrast than of comparison. The one work exhibits a calm, classical, and reflective mind; the other, a busy, clever, and suggestive fancy. The one is to be read to day; the other occupies a permanent place on our library shelves. Each is useful in its sphere and vocation; the part of the critical student will be to extract valuable matter from both.

Before proceeding to comment upon the contents of the present volume, it may be as well to say that many of the Articles—or Chapters, as they are inappropriately called—in it have already appeared in print. But, as they are now collected together for the first time, so as to form a substantive work, we have regarded it as a new book, and shall accordingly give it the privilege of an extended notice.

After some very common-placed remarks on Homœopathy and Hydropathy, and a few words on the subject of Medical Reform—for the merest scrap from our author's porte-feuille is deemed worthy of insertion—we come to the *third* Chapter, on *The Physiology of the Nervous System.*

It need scarcely be said that this is chiefly taken up with an exposition of the Reflex functions of the spinal marrow. As there are several other chapters in the volume on the same subject, we have thought it better, in the present article, to omit the consideration of them altogether; in order that we may have an opportunity of blending their contents, not only with each other, but also with those which will doubtless be found in the subsequent volumes of these " Observations and Suggestions." We proceed therefore to something more practical.

*On the Use of the Alcoholic Lotion in Phthisis Pulmonalis.*

This lotion consists of one part of pure alcohol and three parts of water. A piece of soft linen, folded several times, is laid across the upper part of the chest, just below the clavicles; and this is to be wetted, *every five minutes*, with the mixture—used at first tepid, and afterwards cool. " The application of the lotion should be incessant during the day and all waking hours, the dress being light, or even entirely removed, so as to allow of free and rapid evaporation. (!) It is suspended during the night." This is the principal remedy, by which Dr. Hall assures us that he has benefited, or even restored to apparent health, many persons affected with incipient Phthisis—" marked by dulness of sound on percussion, and no doubtful pectoriloquy under the clavicle, hæmoptysis, and disposition to chills, heats, and early morning perspirations." He subsequently tells us that, according to his experience, this remedy possesses a decided power in checking the progress of the deposition and softening of pulmonary tubercles: *its value* he considers to be *extreme.* We need not mention any of the cases adduced by our author to prove his position. What remedy has not its cures to boast of? Few medical readers, we should think, will be willing to receive " these cases as examples" of the efficacy of the alcoholic lotion: Dr. Hall himself seems hesitating what to say about it:—" I would beg to be understood as stating only the fact, that I have witnessed many, very many, cases of incipient phthisis checked by the strenuous application of the alcoholic lotion, and the patients restored to *apparent* health, these cases having been proved to be phthisis by the presence of the physical signs, as well as the morbid symptoms of this dire disease."

Although little disposed to regard the use of a spirituous lotion to the chest as " the most important remedy in this disease (Phthisis) which we possess," we are much in the habit of recommending it, or something like it—a mixture of equal parts of vinegar, eau de Cologne and water—as a useful and agreeable means of relieving the dyspnœa that is always present in a greater or less degree. The best way of using it is to cover the wetted pledget with a piece of oilskin, so that the application need not be renewed above three times in the course of the twenty-four hours.

*On the Motive for the Scarification of the Gums during Dentition.*

We are gravely told, at the commencement of this Chapter, that " the prevailing, I may say the universal, idea on the subject is, that we should lance the gums only when the teeth are ready to pierce through them, and only at the most prominent parts of the gums." Indeed!—are not medical men in the daily habit of freely and deeply lancing the gums when they are hot, swollen and painful, and when the child is restless and feverish, although the teeth are far from the surface? and are not the scarifications usually practised not only upon the upper, but also upon the lateral, parts of the inflamed gums?

Every one knows, as a matter of course, that there is a hyperæmic condition of the dental and gingival vessels, during the evolution of the gums; and that, whenever the local irritation, which is necessarily consequent upon this state, exceeds a certain degree, nothing relieves the little sufferer so much as a tolerably free discharge of blood directly from the gums. If unrelieved, the child becomes more feverish, and the risk of convulsionary or hydrocephalic disease supervening is increased. There is nothing *new* in all this. That the seat of the irritation is deeper than the mere gums will be admitted by all; nor were we aware, as Dr. Hall asserts, that it is generally imagined by medical practitioners, that "the focus," from which the nervous disturbance proceeds, "is the nerves of the mere gums seated over the prominent parts of the teeth." It did not require to adduce the evidence of nurses to prove "that, *before* the teeth actually reach the borders of the gums, they may prove the source of much irritation:" the fact is of daily or rather hourly observation.

The practical conclusion which Dr. Hall labours most energetically to inculcate is, that scarification of the gums should be practised not only more deeply, but also much more frequently, than is usually done. He advises that it should be performed every day, or even twice daily, as long as there is fever or restlessness, or any tendency to spasm or convulsion; nay, he would sometimes continue the use of the remedy, even when these symptoms have ceased, with the view of preventing their return. This ultra-vigorous practice of our author is not likely to be followed by professional men, even although they thereby subject themselves to reproof for not adopting *his* suggestions. The repeated application of one or more leeches immediately behind the ears, along with the constant use of cold to the head, and of diuretic purgatives internally, may generally supersede the necessity for often-renewed scarifications.

We must not forget to mention that he thinks that the vascular condition of the gums during Dentition might be ascertained by means of a thermometer, and that a useful means of diagnosis might be discovered by its adoption! What say our readers to this ingenious hint? The concluding paragraph of this Chapter is so truly Marshall Hall-ian, that we should do injustice to him to omit it.

"I do not pretend, in the above proposition, to have advanced anything new; but in the *locality* chosen for the operation, and in the *promptitude, repetition, perseverance,* and in the *energy and steadiness of purpose* with which I recommend the measure to be adopted,—if these be fully apprehended,—I believe I do propose something *new;* and when I repeat that since I adopted the plan of *effectually* removing *all* irritation in the gums, stomach, and intestines, in cases of crowing and other convulsions of the same nature, early enough, I have not known or seen a fatal case, I am aware that I propose a plan of treatment at once new and *invaluable*. But half measures are of no efficacy." 34.

*On Stridulous Convulsion in Infants.*

"The disposition to this disease seems to consist in a peculiar susceptibility of the excito-motor property of the spinal marrow. The immediate attacks are the result of the action of sources of irritation or excitement of this property. This susceptibility should, if possible, be diminished, and the causes of excitement should be most carefully avoided. These are the *two principles* which must, I believe, guide us in our treatment." 35.

So much for the *proximate;* now for the *inducing,* causes of the disease. These are, Dentition, indigestible food in the stomach, acrid matters in the bowels, external agents—the most obvious and important of which are atmospheric vicissitudes—and lastly, mental emotions, as fright, passion, and so-forth. Dr. Hall makes some very judicious observations on each of these points. We must, however, take exception to one remark which bears on the interesting subject of the treatment.

"It frequently happens," says our author, "that in the crowing disease, there is a spasm of the gall-ducts, and the alvine evacuation is as pale as white clay. Nothing removes this state of things so effectually as the repeated use of ample 'lavements.' It has accomplished more than the blue pill, the grey powder, or calomel itself." 35.

First of all, is it not very doubtful whether *mere* Spasm of the gall-ducts is of frequent occurrence? It seems more probable that, in the circumstances alluded to, there is either a positive deficiency of the biliary secretion, or a mechanical obstruction to its free exit from the gall-bladder into the duodenum—in consequence, it may be, of the orifice of the *ductus choledochus* being plugged up with viscid phlegm, or from the pressure of some adjoining viscus. However this may be, we would caution our readers not to attach so much importance to the use of warm water enemata, as Dr. Hall seems to imply in the passage which we have quoted. It is right to mention that he had previously recommended the frequent administration of a grain of calomel or blue pill, *if the secretions be wrong.* Are they not so, when "the alvine evacuation is as pale as white clay." The addition of a grain of Ipecacuan and of two or three grains of the Carbonate of Soda to the dose of the mercurial at bed time, and the exhibition of Castor oil next morning, will be found useful under these circumstances. When the bowels have been sufficiently evacuated, the infusion of Rhubarb or Calumba, to which a little Soda, and a few drops of aromatic spirit of Ammonia and of tincture of Henbane are added, may generally be exhibited, with much advantage, two or three times a day.

The following remarks are well worthy the notice of every medical practitioner.

"In reference to the *morbid susceptibility* of the little patient, it is, I believe, best subdued by the tincture of hyoscyamus and the infusion of the humulus lupulus. The system may be kept constantly under the gentle influence of these remedies; that of the exciting causes is then less injurious. The gentle tonic influence of sponging the general surface with tepid salt water is also highly beneficial. All inclemencies of the weather being avoided—for heat, cold, damp, and the north-easterly winds, are alike injurious; yet the child should be much in the open air. It should be protected, not only by the *shade,* but by a flannel dress which should cover every part of the surface, whilst the clothing in general should be suited to the season." 42.

With respect to the alleged *thymic* origin of Stridulous Dyspnœa, Dr. Hall is of opinion that the enlargement of the thymus gland—a morbid condition that unquestionably does not unfrequently exist in some places —is the *effect*, rather than the *cause*, of the suffocative symptoms.

Without adopting the pathological views of certain German writers on this question, we must confess that we do not think that our author has so satisfactorily proved his position as to entitle him to say that "this case affords another example of morbid anatomy, erroneously interpreted, leading to erroneous views of disease."

*On the Use of Setons in certain Diseases.*

"In a variety of cases of acute or chronic, local or limited internal inflammation, I have had recourse to the seton, and uniformly with the most marked success; so that, I think, we may look upon the remedy as almost specific in such cases. It is unnecessary to enumerate them. But hepatitis and nephritis belong to them in an especial manner, and I would suggest this remedy as likely to be of service (if any remedy can) in the case of albuminous urine. In one such case, the urine was more albuminous after cupping. I imagined the effect arose from the mechanical violence inflicted, and recommended the cupping to be performed above and below the precise region of the kidneys. Under the use of this remedy, the albumen diminished, and even ceased for a time." 48.

No one, who has seen much of medical practice, will dispute the great remedial powers of artificial drains in numerous cases of obstinate chronic inflammation, and—not unfrequently also—of incipient organic disease. Thus in Paraplegia, arising from disease of the Spinal Marrow, they will often produce very salutary effects. As a matter of course—and surely this point of practice is perfectly well understood by all enlightened practitioners, notwithstanding Dr. Hall's insinuation to the contrary—in such cases they should always be inserted along the spine, as near to the roots of those spinal nerves that are affected, and therefore *above* the transverse boundary of the paralysed parts. He recommends that as many as four or even six setons should be inserted in some cases of Paraplegia. Few patients will submit to such an infliction, nor can we understand the necessity of this excessively active—*new*, it certainly is—treatment under such circumstances.

*On Cupping.*

Is there any thing novel in the following observations?

"It frequently occurs to the physician to wish to relieve an internal pain or other affection without inducing exhaustion—to relieve the head, for example, without depleting the general system.

"I have found two modes useful in these circumstances: the first is the application of the cupping instrument *twice*, so as to make incisions crossed at right angles, applying the cupping glasses slightly, so as to take very little blood; the second is the application of the cupping glasses alone, commonly called dry-cupping.

"The object of the first mode of proceeding is to induce effectual counter-irritation. The crossed incisions, which may be repeated, become inflamed; or they may be excited to inflammation by proper applications; and in this manner they act as minute temporary issues. Issues and setons themselves are, I believe, more efficacious, at the first, when inflamed, than afterwards, when they merely pour forth a purulent discharge." 51.

If the last remark be true, would not repeated blistering be more useful than either of these means ?—and yet such is not the case in many instances of chronic disease.

The suggestions in the following Chapter, *On the Treatment of Lateral Curvature of the Spine,* are all very ingenious; but have they ever been applied in practice ? We fancy not.

Next come some clever remarks on the best means of keeping up a regulated temperature and moisture in a sick-room. The following sentence stands out detached and by itself—to mark its importance, we presume.

"I have frequently thought that boiling water might be made to circulate from the boiler in the kitchen, through a water-stove in the room or rooms above, so as to supply temperature without additional fires." 63.

We pass on to a somewhat kindred subject.

*On the Exclusion of the Atmospheric Air in the Treatment of certain Diseases.*

"It is usual, in the Parisian hospitals, to trust the treatment of pleuritis greatly to the application of cataplasms. I confess that, when I first heard of this mode of treatment, I thought it trifling. I have since thought that these cataplasms may entirely exclude the influence of the atmospheric air, and prove of real efficacy.

"But whatever may be the rationale, the fact remains as I have stated it; and where the treatment of pleuritis consists greatly in the application of mere cataplasms, a post-mortem examination in this disease is scarcely or not to be obtained, so generally do the patients recover." 68.

Truly this remark savours strongly of the practice of M. Louis; who made the important discovery, some years ago, that bleeding and blistering have little or no effect on the cure of Pneumonia! Dr. Hall appears to be quite smitten with the idea; for he proceeds to remark that "it is, probably, by the exclusion of the atmospheric air that other remedies for inflammatory diseases act; the various plasters, the nitrate of silver, even blisters, have this effect. I do not, however, mean to insinuate that they have no other." Nay, more than this, he alludes to a case of Scirrhus of the Mammæ, in which a mild adhesive plaster was applied to the tumor. It remained adherent for years (the same plaster ?), and the disease continued stationary. It then separated, and from that period, the disease pursued its devastating progress. Would that we dare hope for such a result from so simple a remedy!

In his observations on

*The Use of Enemata of Warm Water,*

Dr. Hall strongly recommends them in Icterus, (we have already alluded to his opinion on this point of practice), in Dysmenorrhœa, and "in cases of intestinal load and irritation, inducing sickness and vomiting, sick headache, sick epilepsy (if I may use this term to designate an attack of sickness, faintishness, perspiration, and an epilepsoid attack), the various fits of children, &c." In such cases, no remedy is so *prompt,* he says, as a large warm water injection. He very judiciously adds that the act of vomiting also is usually very serviceable. In infantile convulsions, the gums should be freely lanced,—and in Dysmenorrhœa, the uterine region should be assiduously fomented—at the same time.

*On the Prevention of Milk-Abscess and Milk-Fever.*

Regarding a mammary abscess as consisting originally in a distended condition of the milk ducts, our author very reasonably concludes, that the prevention of the evil will be most effectually promoted by carefully and assiduously *drawing* the breasts, so that there is never any accumulation of the milk in the lactiferous tubes. If, however, the following advice be intended as a universal or even general rule of practice, we must beg leave to take exception to it.

"As a preventive of milk-abscess and milk-fever, and with other hygienic objects, the infant should be put to the mammæ at the moment it is born. If, in spite of this, the mammæ become in the slightest degree tumid, or febrile action be set up, another and a stronger infant should be applied *without delay.*" 75.

To apply a child to the breast, the moment after it is born, is a piece of advice that savours more of "*promptitude, energy, and decision of purpose*" (the Italics are the author's), than of practical acquaintance with "*Nature's* mode of relief." The milk is rarely formed within from twelve to twenty-four hours after delivery; and we need not say that, as a general direction, nothing should be done to *urge* the secretion. When this is once established, we quite agree with our author—and indeed with every other rational writer on the management of lying-in women—as to the necessity of keeping the mammæ well *drawn,* if there be any disposition to the formation of a milk-abscess. The claim of our author, to having proposed something *new* on this occasion, is really most amusing.

*Chap.* XV.—*On the Causes and Prevention of Apoplexy and Paralysis,* is one of the most valuable in the book. Dr. Hall describes, with much practical acumen, the various states of the system in which the symptoms, that indicate an impending attack of these diseases, are apt to occur; and he points out, at the same time, how very different should be the treatment that is pursued in different cases. In one case, blood must be drawn promptly and to a large extent; while, in another, all depletion should be carefully avoided, and we should trust to rest and quietude, the application of counter-irritants to various parts of the body, and perhaps also to the internal use of ammonia and other stimulants—so as to restore the equilibrium of the circulation. The tact of the judicious practitioner lies in discriminating the two sets of cases—whose symptoms, be it remembered, are often perplexingly alike—and accommodating his plan of treatment accordingly. The too common practice of at once whipping out a lancet, and opening a vein in the arm of a person who is threatened with a fit, or has already fallen down, cannot be too severely reprobated. Many a patient has lost his life, because a *doctor* bled him. The reason of this is obvious:—there is often more of *syncope,* than of vascular congestion of the cerebrum, in a *seeming* apoplectic seizure.

In ambiguous cases, what is the medical man to do? Our author says:

"There is a resource in such a case, which, in spite of a criticism in a very respectable author, I will again venture to assert, is of immense value, and to which I shall have to revert in some subsequent chapters. There is no case in which the patient, if bled from a good orifice, in the erect posture, bears to lose so much blood before syncope takes place, as that of real congestion of the cerebral vessels; there is no case in which the full, not to say the lavish, detraction of blood is so urgently necessary. On the other hand, the case of vertigo, and other symptoms of cerebral affection arising from dyspepsia, neither bears the loss of much blood, taken under similar circumstances of posture, &c. nor requires it.

"In a doubtful case, I propose to adopt this mode of blood-letting; first, as a guard at once against the inefficient and the undue loss of blood, and, secondly, as a DIAGNOSIS, and as a prompter of our ulterior proceedings." 80.

We should prefer having recourse, under such circumstances, to cupping, and the use of a stimulating purgative enema. The *effect* of these means will enable the physician to determine whether more copious depletion is advisable.

Dr. Hall's remarks on the *gouty* and *dyspeptic* forms of apoplectic and paralytic disease are exceedingly good, and will well repay perusal. He has omitted to mention that, in the former variety, there is generally a cretaceous state of some of the arteries of the brain or of the heart; or, it may be, of both viscera in the same individual.

Under the name of "*Temper Disease,*" our author describes various forms of Neuropathy—a morbid condition, in which, it is well known, there is so often some strange perversion of the will and temper, at the same time. They are usually either of a neuralgic character, with or without a spasmodic action of certain muscles, or of a paralytic one. The patient is always of a more or less Hysterical or Hypochondriacal disposition. Most cases occur in the female sex, and chiefly among young and highly excitable girls. One loses her speech; another the use of her legs; a third cannot swallow; and a fourth vomits all, or some particular article of, her food. Retention of the urine is not an unfrequent symptom; and abdominal pain and tympanitis are almost invariably present, in a greater or less degree. Dr. Hall vouches for the authenticity of the following case; otherwise we should have taken it for one of the cleverly-told stories in the "Diary of a Physician."

"A young person of hysteric disposition was bled, and soon afterwards became affected with contraction of the fingers into the palm of the hand. Under the idea that the nerve had been wounded, the cicatrix left by the venæsection was removed: the spasmodic action of the fingers immediately became relaxed, and their use was restored. By degrees the spasm returned, and the operation was repeated with the same good effect, less prompt but not less perfect than before. The spasm returned a third time.

"I now began to suspect that even this strange degree of spasm, during which the nails actually grew into the palm of the hand, was not altogether real. I suggested that the patient should be blindfolded, and that a mock operation should be performed. It was performed: superficial but painful lacerations were made in the integuments: it was pretended that a nerve was laid bare, was divided; and it was loudly said, 'Now the spasm will cease, and she will open her hand;' and she did open her hand! Water was coloured with the tinctura lavandulæ composita, for the want of blood! Again, after a time, the spasm seemed to be returning; but now the whole truth was told; and the patient, for fear of exposure, took care to remain well." 94.

The chapter on *Aphoria*, or *Sterility*, contains some very ingenious, but

(we fear) not very practicable, suggestions. After alluding to various phenomena—some of them of much interest*—that prove the close sympathy that exists between the Mammæ and the Uterus, Dr. Hall throws out the following hint.

" My suggestion then is, that when the mamma is excited at the return of the catamenial period, a robust infant be repeatedly and perseveringly applied, in the hope that the secretion of milk may be excited, and that the uterine blood may be diverted from the uterus and directed into the mammary vessels, and that a change in the uterine system and a proneness to conception may be induced.

" To show that such an event is not improbable, I here quote a singular remark of Dr. Heberden :—' Fœminæ quadragenariæ mammæ cœperunt tumere, et mox lacte impletæ sunt, quod per tres menses exstillabat; protinus vero ut lac in mammas fluere desiisset, mulier hæc e viro suo concepit, quæ per sex annos gravida non fuerat.' "

" I would propose, then, that the patient should sleep, for one week before and during each catamenial period, with an infant on her bosom." 157.

Our author, in his suggestive zeal, makes another proposal, which,—to say the least of it—is rather a droll one. From the well-known effects of a *douche* of cold water on the hypogastrium in exciting contraction of the uterus in cases of flooding, he thinks that " its due administration immediately after intercourse" might be effectual in obviating that " inertia of the uterine system on which atonic sterility sometimes depends. The actions, which lead to conception, may be of the excited class. Contraction of the uterus may be excited, and be followed by relaxation and ingurgitation. I need only make the observation. The inference is obvious." Who is the medical man that will first make trial of this *new* remedy ?

The *Cursory Remarks on Prognosis* in Chapter XXV. are admirable. Their object is to shew that, in various maladies, there is apt to supervene, ***most unexpectedly***, a rapid and often fatal sinking of the powers of life. In diseases of the brain or of the heart, after great losses of blood, or excessive purgation, after parturition in weak delicate habits, &c. this most dis-

---

* The following passage from Dr. Rigby's System of Midwifery—published as the sixth volume of Dr. Tweedie's Library of Medicine—cannot be too generally known.

" The application of the child to the breast is not less valuable for preventing any return of the hæmorrhage, than for stopping it in the first instance. We are *never* perfectly secure against hæmorrhage coming on during the first few hours after delivery, even where every thing has turned out as favourably as possible; the exhaustion from the length or severity of the labour, the warmth of the bed, and, in some cases, it would seem, the relaxing effects of deep sleep, are all liable to be followed by inertia uteri and hæmorrhage. In no way can we insure our patient so completely against this kind of danger as by putting the child to the breast. The uterine contraction which it excites is not only powerful, but permanent; nor do we consider that a practitioner is justified in leaving a patient in whom the uterus has shewn a disposition to inertia without having insured her safety by this simple, but effectual safeguard. 153.

tressing occurrence has repeatedly been observed to take place. "But the most treacherous cases," says our author, "are those of intestinal obstruction, from various causes. The patient shall have suffered from sickness,—rejecting all food and medicine; and from constipation, resisting every kind of aperient or purgative, however administered, whether swallowed, injected as an enema, or applied to the tongue (croton oil), or endermically: at length the bowels shall be moved satisfactorily, and the patient shall appear better in every respect! The appearances are fallacious. The powers of the heart (so apt to be impressed through the medium of the stomach and bowels) shall have yielded to the struggle; a state of the most insidious *sinking* has set in. Food is retained; the bowels are freely moved; all pain is gone; the patient, as I have said, appears better in every respect; but, in the course of the night, or in the course of twenty-four hours, sinks and expires."

Dr. Hall points out certain symptoms which often precede this state of exhaustion, and the observation of which, in due time, may lead the practitioner to adopt measures for its prevention. These are *breathlessness* upon the least exertion—*a slight crepitus in the breathing*—*a tympanitic tumefaction of the abdomen*—and *a peculiar severe pain on one side of the neck.*

A very different character we must give of the *Cursory Remarks on Diagnosis,* in Chapter XXXVI.; for, in truth, they are little more than an advertisement that the indefatigable author hopes, "in due time, to lay before the profession a treatise on Diagnosis, in all its useful and practical bearings, not unworthy of the present advanced state of medical science and practice." To prevent every possible mistake, this announcement is given twice in the course of the volume.

Dr. Hall's *Treatment of Chronic Bronchitis, Chronic Pneumonia,* &c., is not likely, we should think, to become popular either with patient or physician. In one case, "*for three whole years* (the Italics are our's), a sharp liniment was applied over the back and front parts of the thorax, night and morning, without intermission. The result was, that the chronic Bronchitis was effectually cured." A reward indeed of diligence, and "of the steady perseverance that may be required in such cases!"

Besides the use of counter-irritants, we should cautiously "exclude the influence of the oxygen, dryness and temperature of the atmospheric air, on the *external* surface of the thorax." This is most effectually done, we are told, by covering the entire chest with "an ample adhesive plaster": the patient to wear, as a matter of course, a waistcoat of flannel, silk or leather, and to keep the feet warm and dry.

"But the most important part of the treatment is that to be pursued during the night. I have frequently found the following plan of extreme value: a sort of mosquito net is formed of muslin; this is made to pass over and enclose a chair, on which is placed a large jar nearly full of water, at 180° Faht.; and under the same net the patient sleeps. During the whole of the night, he inhales a warm and genial vapour, whilst his face is exposed to it, and the whole surface is influenced by it. A state of the skin, and of the air-tubes and cells, is induced which is very favorable to the cure of chronic inflammation within the chest.

"The success of this plan in chronic bronchitis, pneumonia, and pleuritis, has been most gratifying." 203.

Dr. Hall thinks so highly of this ingenious suggestion, that he has described it in two different chapters. By the bye, he appears to be a great friend of the Arnott stove for warming sick rooms.

The whole of Chapter XXXIV. is taken up with the details of *a case* (sufficiently common in practice) *of feverish or inflammatory Cold, Otitis, Brown-Ague*, &c. If there was " extremely severe Gastritis" present, how comes it to pass that we hear nothing of leeches being applied to the epigastrium ? Altogether the narrative is not worthy of the space which it occupies. The little patient seems to have been a pet child. Among other interesting particulars related, we are informed that one evening he " was moved to the sofa, and had his feet put into hot water, whilst his bed was freshened. He took some tea, gradually fell asleep, slept well, and awoke free from fever, and with an appetite."

*Chapter* XL., *On the Prevention of Insidious Disease of the Brain in Children* is, for more reasons than one, most unsatisfactory—to the professional reader at least : we cannot answer for the tastes of " the general reader, who may be interested in medical matters generally, or in some particular medical subject," to whom the present volume is partly addressed. Reference is made to two cases, in which Dr. Hall, very unnecessarily, intimates that " the medical attendants had been taken by surprise, by the insidious development, progress, and termination of this dire disease (Hydrocephalus) of children ; and the question was—how could such an event be averted in regard to the other children ?"

Part of the correspondence that took place on these occasions, is given. The first letter is from our author to ———, Esq. M.P., and contains an elaborate account of the *regime* which the doctor advised should be followed out in the case of certain young members of his family. Among other injunctions, he recommends that " a *little mutton* should be taken *thrice* a day,—at breakfast, dinner, and tea ; and with this, *very little* vegetable food, the latter consisting of stale white bread (five days old, and exposed so as to dry into a sort of biscuit), well-cooked rice, and a perfectly mealy potatoe."........" The shoes should be changed, be this needed or not, for others perfectly warm and dry, four times a day ; and oftener if necessary."........" Avoid calomel, blue pill, &c. (if possible) as *cane pejus et angue* as well as all lowering remedies." After very properly enjoining that the bowels should be freely relieved once every day, the punctiliously exact doctor gives the following see-saw medicinal directions :—

" I next advise five, seven, or ten drops of steel wine to be given thrice a-day, in a table-spoonful of water, in the midst of meals, for one month ; then half, two-thirds, and one grain of the sulphate of quinine, in the form of pill, thrice a-day at meals, for another month, omitting the steel wine ; then *both* these medicines for a third month. I would then give one, one and a half, two, and two and a half wine-glassfuls of the pale ale prepared for India, for a fourth month, omitting the tonic medicines. Then return to the steel wine, the quinine, and both, as before, and so on." 240.

How comes it that no notice is taken of a very important part of the *regime* in such cases—that of not allowing the hair to grow long (more especially in girls), and of keeping the head cool by frequently wetting it with water, or any simple spirituous wash ?

We know not what to make of the other letters that are contained in this chapter. They are addressed to Mr. ——— ; but whether he be a medical man, or only an *amateur-medecin*, we cannot make out. Certain it is that he writes very learnedly about what he had heard respecting an inflammatory action of the Brain in Hydrocephalus, and about the system of treatment which he had been accustomed to contemplate ; nay, even he gives his opinion touching the necroscopic appearances found in the head of another child whom he had lost. But then the rest of his epistle seems to imply that he is not a regular medical man ; the closing paragraph is strange ; " I owe you an apology, I think, for almost writing for an essay on Hydrocephalus, as well as for the treatment of it ; but I feel sure you will see my position ; and I cannot act in the dark, knowing what little I do."

There are several points in Dr. Hall's reply that deserve brief notice. First of all, he deems it " very questionable whether such a *low* form of inflammation, as that alluded to, really exists in young and not apparently weakly subjects." That a subacute inflammatory action of the Encephalon is present in many cases of Hydrocephalus, for a length of time before the outbreak of the pathognomonic symptoms, will be admitted, we should think, by all practical men.

With respect to the use of *mercurials*, Dr. Hall appears to be (as we have already seen) decidedly adverse to their employment ; grounding his objections partly on their *lowering* effects upon the system, and partly also because, in his opinion, the intestinal secretions may be corrected without them—nothing being so effectual, he says, for this purpose and for inducing a flow of *bile*, as the warm-water enema.

The concluding paragraph merits an attentive examination. Like most other doctrines in medical practice, the one laid down here is to be received not so much as an absolute truth as in the light of a useful admonition ; a great deal must be left, in every case, to the discretion of the medical attendant.

> " You must constantly bear in mind the difference between the *preventive* and the actually *curative* treatment in these cases ; they are so different as to be almost opposite, both in their character and in their measure. This remark will tend to reconcile what may appear to you to be a glaring discrepancy in the medical opinions which have been given in the cases of your little patients." 253.

In Chapter XLI. are briefly related two or three cases of *Hydrocephalus* —or rather the intense Cerebral Congestion that sometimes constitutes its first stage—proving fatal within twelve and even six hours after the attack. Convulsions are usually the most striking and formidable symptom of this most alarming form of the disease. It is apt to occur during recovery from Scarlatina, especially if the urinary secretion has remained scanty and high-coloured ; but it may take place under other circumstances ; and a physician, therefore, cannot be too much on his guard against its invasion. Prompt and copious bloodletting is the most important remedy ; the use of this potent remedy must be followed by mercurials, active diuretic purges, and the assiduous application of cold to the head.

The remarks, in Chapter XLIII., on *Tuberculous Disease of the Abdomen*, are in one respect good; in another faulty, inasmuch as they direct the attention of the reader almost exclusively to the abdominal malady, without attaching due importance to the co-existent affection of the lungs. Tubercles are rarely found—at least, to any considerable extent—within the abdomen, unaccompanied by similar depositions in the pulmonary tissue. In the only one of Dr. Hall's cases, in which a post-mortem examination took place, we read that " the right lung contained tubercles, some of which were softened and suppurated; and the left lung was heavy, fœtid, and replete with tubercles and partial suppuration." The case might therefore be regarded as one of Phthisis, attended with tubercular disease of the abdomen. Dr. Hall dwells with great emphasis on three symptoms, which he regards as characteristic of this disease—*great tendency to coldness and lividity of the extreme parts of the body, a frequent pulse, and slow but progressive emaciation.* The existence of these symptoms should therefore always draw the attention of the physician to the state of the abdomen, and, we need not add, of the lungs, at the same time.

In the Chapter on *the Effects* of *Intestinal Irritation*, Dr. Hall seeks to shew that a morbid condition of the bowels, arising from an accumulation of fæculent matters or from a depraved state of the intestinal secretions, is apt to induce sudden attacks of severe pain, accompanied with the other symptoms of active inflammatory disease, either in the Brain, the Pleura, or in the Abdominal Viscera—but which attacks are in truth rather of a sympathetic and nervous, than of a really phlogistic, character. We need not say how important it must always be to form an accurate Diagnosis in such cases; seeing that the Treatment will, as a matter of course, depend on the opinion that has been formed by the medical attendant as to the nature of the *seemingly*-inflammatory attack. The practical question may be between bleeding to 20 or 30 ounces, and the exhibition of an ammoniacal opiate draught, to allay the pain and other symptoms.

That Dr. Hall has considerably exaggerated the difficulties of an accurate diagnosis, we shall attempt to prove by an examination of his own cases. A middle-aged and rather delicate woman had been very largely bled and freely purged for what appeared to be an attack of Peritonitis: the stools were very fœtid. She seemed to be recovering fast, when, on the fourth day, she was " seized with severe pain of the head, especially over the eyebrows, attended by beating and throbbing, and by the most urgent intolerance of light, so that the eyes could not be opened for a moment for examination; the pain was increased on attempting to sit up erect; the countenance was palish and sallow; the pulse full and frequent; there was no faintness or sighing."

A draught, containing 30 drops of laudanum and as many of Sal Volatile, was administered, and a cold lotion applied to the head. Next day, the patient was much better; and speedily she was quite well. Dr. H. proceeds to say that here was a case, where the symptoms, " usually deemed indicative of Phrenitis *in its most marked form*," were removed, without the lancet, by an ammoniacal anodyne draught! We much doubt whether any experienced practitioner could ever have mistaken the attack of severe *headache* in this case for one of *inflammation of the brain:* the mere circumstance of the pain being increased in the erect position might have led, we should think, to a more correct diagnosis.

The next case is still more unsatisfactory; for the patient—who was at the time in a restless irritable state from pain and want of sleep for several nights—seems to have been bled for mere *throbbing in his temples, with headache, and flying stitches in his side*, although all the other symptoms clearly denote positive weakness, rather than the existence of any inflammatory action. Judge our surprize therefore when we find, in Dr. Hall's comments on the case, these words: "I think most physicians would have apprehended, at least, some inflammatory affection within the head." Not so, we believe, good doctor; do read the report of the case over again.

The third case seems to have been one of very flagrant mal-practice. The symptoms, as recorded, did not surely warrant the excessive depletions that were employed. The patient, a female, was six times bled; and yet no mention is made on any occasion, either of the state of her pulse or of that of the blood drawn. Suffice it to say that, when Dr. Hall was called in, "there was palpitation of the heart, and sometimes faintness, and a feeling of sinking or dying." By administering cordials and nourishment, and correcting the state of the bowels that were much disordered, this patient quickly recovered.

Case 5 is one of every-day occurrence; the attack was nothing but one of bilious or dyspeptic *headache*, and accordingly was relieved very promptly by vomiting and a dose of purging physic. In case 6, we have an example of *pleurodyny*, and in case 7 one of *palpitations* of the heart. As the report of the last is very brief, we shall give it entire, to convince our readers that our ingenious author has somewhat over-strained the point which he seeks to establish in this chapter.

"In this case, of which the heads only can be given, the patient was afflicted with great *palpitation of the heart*, which returned in paroxysms. The attack would come on from various causes, induce great alarm and sense of dissolution, with throbbing along the abdominal aorta. The patient was bled profusely, without more than temporary relief. He recovered gradually under the employment of purgative medicine, nutritious diet, and soothing treatment, remaining only subject to dyspepsia." 300.

No treatment could possibly have been worse than profuse blood-letting in a case of paroxysmal palpitations of the heart: a dose of laudanum and æther would, as a matter of course, have been much more appropriate.

We do not by these strictures mean to deny the position of Dr. Hall, that intestinal irritation is liable to be accompanied with attacks of severe pain and febrile excitement in one or more of the internal cavities of the body, and that these attacks may occasionally exhibit many of the characters of active inflammatory action, so as to be apt to mislead the inexperienced practitioner. All that we maintain is, that, in the cases related by him, there ought, we think, to have been little or no uncertainty experienced as to the correct diagnosis. How unsatisfactorily is the distinction between actual and *seeming* inflammatory affections of the abdomen made out in the following passage!

"I had long remarked that there might be both acute pain, and tenderness under pressure, of the abdomen, without inflammation. This state of things is frequently the result of intestinal irritation. It is distinguished from inflammation by the *general* symptoms of this affection,—the mode of attack,—the effects of remedies. In inflammation, the surface is usually cool, the head unaffected, the patient remarkably quiet. In the case of intestinal irritation, on the contrary, there is generally much heat after rigor, the head is much affected, and the patient is restless and generally distressed, the tongue is loaded, and perhaps swollen, the alvine evacuations are extremely morbid, and great relief is obtained by the free operation of medicine." 304.

Altogether, this chapter is not at all worthy of the author's well-known sagacity.

The following one, *On the State of Sinking from various Causes*, contains many valuable observations, derived partly from the author's own experience, and partly from the writings of John Hunter and Sir H. Halford. In infants and young children, this state is apt to occur in the course of cerebral and abdominal affections, after bleeding or purging has been used very actively, or continued for some time. Whenever the surface of the body or extremities become chilly, the cheeks are pale and somewhat sunken, the pulse is rapid and weak, and the child is restless and irritable at one time, and dozing and oppressed at another, it is high time to suspend the use of all lowering remedies, and to have immediate recourse to light nourishing food, gentle cordials and stimulants—brandy and ammonia are usually the best—and the assiduous application of warmth to the body and feet. The patient, under such circumstances, should be visited frequently, so that every change in the symptoms may be watched, and the administration of remedies may be regulated accordingly. As calm sleep is one of the most important restoratives, it may be necessary to give two or three drops of laudanum or of the liquor opii sed. in some aromatic water. The continuance of cold applications to the head, after the active cerebral symptoms have subsided, is apt to induce restlessness and discomfort; and we have often observed that, by merely ceasing this application and covering the head with a night-cap, a refreshing sleep has come on, and the young patient has awoke in every respect better.

Passing over the remarks on *Sinking in Old Age*, which Sir H. Halford has very graphically described in his Essay on the Climacteric Disease, we come to the third section of the chapter, that treats of *Sinking in certain Diseases*. The late President of the College of Physicians wrote an able paper on this subject also, entitled the "Necessity of Caution of the Estimation of Symptoms in the Last Stages of some Diseases."

*Phrenitis, Enteritis, Hernia*—even after the bowel has been reduced, but before the vomiting has ceased and the bowels have acted—confluent *Variola*, may be mentioned as diseases, in which a fatal sinking is not unapt to occur, at a time when the patient seems to have surmounted the dangerous part of his illness. After quoting some of Sir Henry's observations, our author remarks:

"The diseases in which the state of sinking is most marked, are, I think, Typhus Fever and Enteritis, Dysentery, or Cholera; though many other diseases lead to this state, and especially some which consist in repeated attacks, each attack leaving the patient weaker than before, until they issue in sinking of the vital powers.

"Amongst the first symptoms, coldness and lividity of the hands are frequently observed, the livid colour disappearing imperfectly on pressure; the cheeks and nose are at the same time usually cool. There are often much general and indefinable suffering, distress, and restlessness; sometimes slight dozing; at others, slight delirium; and in some cases convulsion, followed by coma; the breathing is sometimes imperfect; at others, little affected; and I have, in some cases, observed the crepitus in breathing of which I have spoken in the preceding essay, for some days even before there was any other decided symptom of sinking; the voice is frequently altered, and rather husky; the pulse is small and frequent, and perhaps irregular; the motions are apt to be passed involuntarily, and sometimes there is retention of urine. It is usual for some distressing symptom, as delirium in phrenitis, cough in affections of the chest, and pains in those of the abdomen, to have ceased as the state of sinking has come on." 326.

Laennec has noticed, in the following passages, the tendency that exists to unexpected *Sinking* in certain cases of Pneumonia.

"Chez d'autres sujets, au contraire, la péripneumonie détermine la mort avant que l'engorgement ait envahi le quart de l'organe pulmonaire. Ce fait est propre, ainsi que beaucoup d'autres, à prouver que, dans les altérations nos organes, la mort est souvent due à l'affaiblissement du principe de la vie beaucoup plus qu'à l'intensité ou à l'étendue de l'affection locale."—§ 201.

"On ne voit que trop souvent des exemples de péripneumonies qui, après l'emploi de la saignée et des anti-phlogistiques, paraissent au bout de quelques jours à-peu-près guéries, si l'on s'en rapportait aux symptômes extérieurs: la fièvre a cessé, la douleur n'existe plus, la toux devient rare et peu pénible, l'expectoration est médiocre, les forces renaissent, l'appétit reparait et devient quelquefois même très-vif, et cependant l'engorgement pulmonaire n'a nullement diminué: la percussion donne un so mat, le cylindre ne fait rien entendre. Au bout de quelques jours, et même de quelques semaines d'une fausse convalescence, les forces tombent de nouveau, et, suivant l'âge du malade, un nouvel appareil inflammatoire ou une dyspnée accompagnée d'affaissement et de symptômes de congestion cérébrale sont promptement suivis de la mort."—§ 212.

Chapter XLVIII. contains, as far as we can judge, nothing that is not perfectly well known to every medical man. To tell us that Arthritis or Gout is a malady "of a peculiar *reflex* action," is merely darkening knowledge by words; and that "in cases of a true arthritic nature, by which I mean the same affection interiorly as we observe exteriorly, the Colchicum seems to be indicated," is a piece of therapeutic information that will certainly not be very novel to any of our readers. That the case of retrocedent Gout, related at length by the late Dr. Haygarth, in the 4th volume of the Transactions of the College of Physicians, and given at length in the present volume, was "plainly and simply one of *intestinal irritation*," we have very great doubts, in spite of the very absolute tone in which Dr. Hall settles the question. The very circumstance that the experienced narrator of the case does not even mention that the intestinal evacuations were much disordered—while he alludes more than once very particularly to the high-coloured and sedimentary state of the urine—might surely have made Dr. Hall pause before deciding so peremptorily on the diagnosis of a case which he never saw.

Chapter XLIX. gives the report of an interesting case of *Chronic Laryngitis*, in which the operation of Laryngotomy was deemed necessary, in consequence of the alarming attacks of dyspnœa to which the patient was liable: a cure of the local disease was subsequently effected by the inunction of mercury, so as to affect the system. It is not stated whether the patient—a woman ætat. 53—recovered her voice completely.

Chapters L. and LI. are remarkable only for their brevity; the longest not exceeding 23 lines. The former is entitled, *On a Source of Diagnosis in Laryngitis:* it runs thus:—

"In pure laryngitis, the patient cannot snuff up: see page 342.* The volume of air so admitted into the larynx, though its due velocity be not wanting, is insufficient to produce that effect.

"Laryngitis, in which there is thickening of the lining mucous membrane of the glottis or larynx, and consequent diminution of this orifice, is distinguished in this manner from tuberculous ulceration of the glottis or larynx, in which, so far from there being diminution, there is augmentation of the orifice.

"The effort to 'snuff up,' in laryngitis, has the most peculiar effect. Instead of the *expected* noise in the nostril, there is an *unexpected* sound in the larynx.

"But not only is the *fact* of diminution of the laryngeal orifice ascertained in this manner, but the *degree* of that diminution is marked by the greater or less *degree* in which the power of 'snuffing up' exists, and therefore the greater or less *degree* of urgency of the case! In the same manner, the diminution or augmentation of the disease is accurately marked.

"The same observations have a certain relation to the *double* danger of this disease—from—1, *immediate,* and 2, *secondary,* asphyxia." 349.

The sum and substance of Chapter LI. *On the Treatment* of the *Atrophy of Paralytic Limbs,* by William Frederick Barlow, Esq. are contained in these few lines: "I would suggest...... that Galvanism be used at intervals more or less lengthened as circumstances indicate, or tickling, friction, and temperature be employed, if these be found to occasion reflex actions."

There is surely nothing *new* in this advice, although the writer seems to think otherwise; for he says: "no one, as far as I know, has proposed that *involuntary* contractions should be excited in them (the muscles) *with a view to their nutrition.*" Really if we go on at this rate, there will be nothing *old* under the sun.

With this remark we close our notice of this volume. Our readers will be able to judge for themselves, by the extracts we have given, that it contains much curious and not a little instructive information. We look forward with interest for the succeeding volume or volumes, which are promised, and trust that the talented author will, for the sake of his own reputation, render them more free from the blemishes which we have pointed out.

---

* Reference is here made to the case narrated in the preceding Chapter. ......"She described the impossibility of snuffing up the nostrils—an effect, I suppose, of the partial closure of the larynx; for, to produce this snuffing, it is necessary that a certain *quantity* of air should be drawn through the nostrils with a certain *velocity;* and, in the present instance, the *quantity* of air admitted appears to have been too small. The patient experienced increased uneasiness on drawing the head backwards."

